# A prospective study of endothelial activation biomarkers, including plasma angiopoietin-1 and angiopoietin-2, in Kenyan women initiating antiretroviral therapy

**DOI:** 10.1186/1471-2334-13-263

**Published:** 2013-06-04

**Authors:** Susan M Graham, Nimerta Rajwans, Kenneth A Tapia, Walter Jaoko, Benson BA Estambale, R Scott McClelland, Julie Overbaugh, W Conrad Liles

**Affiliations:** 1 Department of Medicine, University of Washington, Seattle, WA 98195, USA; 2 Department of Global Health, University of Washington, Seattle, WA 98195, USA; 3 Department of Medical Microbiology, University of Nairobi, Nairobi, Kenya; 4 S.A. Rotman Laboratories, McLaughlin-Rotman Centre for Global Health, Toronto General Hospital-University Health Network, University of Toronto, Toronto, Ontario, M5G 2C4, Canada; 5 University of Nairobi Institute of Tropical and Infectious Diseases (UNITID), College of Health Sciences, University of Nairobi, Nairobi, Kenya; 6 Department of Epidemiology, University of Washington, Seattle, WA 98195, USA; 7 Division of Human Biology, Fred Hutchinson Cancer Research Center, Seattle, WA, 98109, USA; 8 Division of Infectious Diseases, Department of Medicine, University of Toronto, Toronto, Ontario, M5G 2C4, Canada

**Keywords:** HIV-1, HAART, ICAM-1, VCAM-1, E-selectin, Angiopoietin-1, Angiopoietin-2, Endothelium

## Abstract

**Background:**

HIV-1-related inflammation is associated with increased levels of biomarkers of vascular adhesion and endothelial activation, and may increase production of the inflammatory protein angiopoietin-2 (ANG-2), an adverse prognostic biomarker in severe systemic infection. We hypothesized that antiretroviral therapy (ART) initiation would decrease endothelial activation, reducing plasma levels of ANG-2.

**Methods:**

Antiretroviral-naïve Kenyan women with advanced HIV infection were followed prospectively. Endothelial activation biomarkers including soluble intercellular adhesion molecule-1 (ICAM-1), vascular adhesion molecule-1 (VCAM-1), and E-selectin, and plasma ANG-2 and angiopoietin-1 (ANG-1) were tested in stored plasma samples from 0, 6, and 12 months after ART initiation. We used Wilcoxon matched-pairs signed rank tests to compare endothelial activation biomarkers across time-points, generalized estimating equations to analyze associations with change in log_10_-transformed biomarkers after ART initiation, and Cox proportional-hazards regression to analyze associations with mortality.

**Results:**

The 102 HIV-1-seropositive women studied had advanced infection (median CD4 count, 124 cells/μL). Soluble ICAM-1 and plasma ANG-2 levels decreased at both time-points after ART initiation, with concomitant increases in the beneficial protein ANG-1. Higher ANG-2 levels after ART initiation were associated with higher plasma HIV-1 RNA, oral contraceptive pill use, pregnancy, severe malnutrition, and tuberculosis. Baseline ANG-2 levels were higher among five women who died after ART initiation than among women who did not (median 2.85 ng/mL [inter-quartile range (IQR) 2.47–5.74 ng/mL] versus median 1.32 ng/mL [IQR 0.35–2.18 ng/mL], p = 0.01). Both soluble ICAM-1 and plasma ANG-2 levels predicted mortality after ART initiation.

**Conclusions:**

Biomarkers of endothelial activation decreased after ART initiation in women with advanced HIV-1 infection. Changes in plasma ANG-2 were associated with HIV-1 RNA levels over 12 months of follow-up. Soluble ICAM-1 and plasma ANG-2 levels represent potential biomarkers for adverse outcomes in advanced HIV-1 infection.

## Background

HIV-1-infected persons are at increased risk not only for AIDS-defining infections and malignancies, but also for cardiovascular disease, kidney and liver disease, and non-AIDS-defining malignancies [1]. Increasing evidence suggests that non-AIDS-defining complications are associated with chronic activation of inflammatory and coagulation pathways [2-6]. Because even prolonged effective antiretroviral therapy (ART) may not normalize biomarkers of inflammation and coagulation [2], a better understanding of the relationship between inflammatory pathways and HIV-1 pathogenesis is needed. In particular, the pathogenesis of HIV-1-related endothelial activation merits further investigation [7].

HIV-1-induced inflammation has been linked to increased biomarkers of endothelial activation, including soluble intercellular adhesion molecule-1 (ICAM-1), vascular adhesion molecule-1 (VCAM-1), and E-selectin [5,8,9]. HIV-1 induces up-regulation of these cellular adhesion molecules, leading to an increase in their soluble forms in plasma [5,8,9]. Soluble VCAM-1 and ICAM-1 levels decrease after ART initiation and increase after treatment interruption [10,11]. Higher levels of soluble ICAM-1 have been associated with increased mortality in HIV-1-infected persons [12]. We hypothesized that the extent of HIV-1-related endothelial activation may be associated with altered levels of the vascular growth factors angiopoietin-1 and-2 (ANG-1 and ANG-2), recently identified as important regulators of endothelial quiescence and activation.

ANG-1 is widely expressed in human endothelial cells, where it plays a key role in maintaining vascular stability by binding to Tie-2, a receptor tyrosine kinase [13]. Its effect is antagonized by ANG-2, which blocks ANG-1/Tie-2 binding and is up-regulated at sites of vascular injury or local inflammation [13]. ANG-2 sensitizes the endothelium to tumor necrosis factor alpha (TNF-α), amplifying its effects and mediating pro-inflammatory and angiogenic effects that can result in manifestations of severe systemic infection, including vascular leak and organ dysfunction [13-15]. While plasma levels of ANG-1 are high in healthy individuals, plasma levels of ANG-2 are usually low or undetectable [13-15].

In a study of angiogenic factors and severe bacterial infection in Malawian children, levels of ANG-2 were significantly elevated in HIV-1-seropositive children relative to seronegative children [16]. We hypothesized that advanced HIV-1 infection would be associated with endothelial activation (increased ANG-2, decreased ANG-1) that would resolve, at least partially, after ART initiation. Our aim was therefore to evaluate ART-related changes in ANG-1 and ANG-2 after ART initiation. We also examined levels of soluble ICAM-1, VCAM-1, and E-selectin to confirm that changes in these biomarkers accompanied changes in ANG-1 and ANG-2. A secondary aim was to determine the extent to which angiopoietin levels were associated with HIV-1 RNA suppression and clinical outcomes in this population.

## Methods

### Study population and procedures

The study followed HIV-1-seropositive, non-pregnant Kenyan women who initiated ART in a research clinic in Mombasa, Kenya between February 2005 and January 2008 [17]. Women enrolled if they were eligible for ART according to Kenyan National Guidelines during this period (CD4 count ≤200 cells/ml or AIDS-defining illness) [18], and willing to undergo scheduled follow-up. At treatment initiation and monthly thereafter, women were interviewed using standardized questionnaires about recent behavior and health status, then underwent a standardized physical examination. Blood was collected for HIV-1 quantitation at baseline and quarterly thereafter. A standard first-line ART regimen of stavudine or zidovudine, lamivudine, and nevirapine was provided, in accordance with World Health Organization (WHO) and Kenyan National Guidelines at the time of study participation [18].

All participants gave written informed consent. Ethical review committees of the Kenyatta National Hosipital, Kenya Medical Research Institute, University of Washington, and Fred Hutchinson Cancer Research Center approved the research protocols.

### Laboratory methods

HIV-1 serostatus was evaluated using the Detect HIV1/2 ELISA (BioChem Immunosystems) confirmed by a second ELISA (Recombigen, Cambridge Biotech or Vironostika HIV-1 Uni-Form II Ag/Ab, bioMérieux). CD4 cell counts were determined using FACS Count (Becton Dickinson). Pregnancy testing was performed using a rapid β–human chorionic gonadotropin test (Plasmatec Laboratory Products). Plasma specimens were frozen at −70°C until shipment to Seattle on dry ice or in liquid nitrogen for HIV-1 RNA quantitation using the Gen-Probe HIV-1 viral load assay [19]. The lower limit of quantitation was 100 copies/mL.

Aliquots of stored plasma samples were sent to Toronto on dry ice and stored at −80°C prior to testing for endothelial activation biomarkers [20,21]. Of note, all biomarkers tested are stable for ≥24 hours and resistant to up to four freeze-thaw cycles [22,23]. Plasma concentrations (dilution factors indicated in parentheses) of ANG-1 (1:5), ANG-2 (1:5), soluble ICAM-1 (1:1000), soluble VCAM-1 (1:2000), and soluble E-selectin (1:50) were measured by ELISA (R&D Systems Duoset kits, Minneapolis MN, USA) according to manufacturers’ instructions with modifications: (1) assays were performed in 50 μL per well; (2) plasma samples were incubated overnight at 4°C; and (3) ELISAs were developed using Extravidin®-Alkaline Phosphatase (Sigma-Aldrich Canada Ltd, Oakville, ON, Canada; 1:1000 dilution, 45-minute incubation) followed by addition of p-nitrophenyl phosphate substrate (Sigma-Aldrich Canada Ltd, Oakville, ON, Canada) before optical density reading at 405 nm. Concentrations were interpolated from 4-parameter-fit standard curves. Background levels were determined from blank wells included on each plate (assay buffer added instead of sample), and the subsequent optical density was subtracted from all samples and standards prior to analysis. Samples with optical densities below the lowest detectable standard were assigned the value of that standard. Lower limits of detection for each assay were as follows: ANG-1 19.53 pg/mL, ANG-2 27.34 pg/mL, soluble ICAM-1 3.91 pg/mL, soluble VCAM-1 1.95 pg/mL, and soluble E-selectin 11.72 pg/mL.

### Statistical methods

An intent-to-treat analysis was used, as we were interested in the effect of ART initiation on endothelial activation biomarker levels and the relationship between biomarkers levels and treatment outcomes in all women who started ART. Descriptive statistics were used to present baseline data. Variables for analysis were categorized at clinically meaningful cut-points, if these existed. Karnofsky performance score was classified as asymptomatic or mild symptoms (90–100), moderate symptoms (80), or unable to work (<80). Nutritional status was categorized as adequate (body mass index [BMI] ≥ 18.5), mild or moderate malnutrition (BMI = 16-18.49), or severe malnutrition (BMI < 16) [24]. CD4 cell count was categorized as <200, 200–350, or >350 cells/μL. Suppressed plasma HIV-1 RNA was defined as <100 copies/mL.

Spearman’s rho was used to calculate non-parametric correlations between endothelial activation biomarkers and plasma HIV-1 RNA at baseline. Wilcoxon matched-pairs signed rank tests were used to compare endothelial activation biomarkers among HIV-1-seropositive women at baseline to 6 or 12 months after ART initiation. Generalized estimating equations (GEE) with a logit link, exchangeable correlation matrix, and robust standard errors were used to test for change in detection of plasma ANG-1 and ANG-2 across time-points.

Because additional modeling required normally distributed data, plasma HIV-1 RNA and endothelial activation biomarkers were log_10_-transformed to approximate normality. GEE with an identity link were used to analyze associations with change in log_10_-transformed plasma ANG-1 or ANG-2 at months 6 and 12 after ART initiation, adjusting for baseline values. Predictors included baseline (e.g., age) and time-dependent variables (e.g., CD4 count). The final multivariable model retained factors significant at p < 0.10 in the initial adjusted analysis that also remained significant in multivariable analysis at p < 0.10. Virologic suppression and change in plasma ANG-2 (for the ANG-1 outcome) or change in plasma ANG-1 (for the ANG-2 outcome) were included a priori in both models as time-dependent predictors, to evaluate the relationship with plasma HIV-1 RNA and because the angiopoietins are competitors at the Tie2 receptor, respectively.

Wilcoxon rank sum tests were used to compare baseline plasma levels of endothelial activation biomarkers in women who did or did not die during follow-up. Cox proportional hazards regression was used to analyze associations between endothelial activation biomarkers and other baseline predictors with all-cause mortality after ART initiation. For this analysis, participants were censored at the last visit before loss to follow-up or the month 12 visit, whichever occurred first. All analyses used the intent-to-treat principle, including women who changed or discontinued ART. Data were analyzed using Stata version 11.2 (StataCorp, College Station, Texas). A two-sided p value <0.05 was considered significant.

## Results

### Study population

The 102 participating women had a median age of 36 (interquartile range [IQR] 33–41) and most had advanced HIV-1 infection (17.6% Stage I, 28.4% Stage II, 43.1% Stage III, and 10.8% Stage IV). Two women had brief exposure to first-line ART prior to enrollment (1 day of treatment 1 week before enrollment and 3 days of treatment 4 months before enrollment). All other women were treatment-naïve. The median CD4 cell count was 124 cells/μL (IQR 79–164 cells/μL) and median plasma HIV-1 RNA was 349,066 copies/mL (IQR 149,106–919,173 copies/mL). Nineteen (18.6%) were being treated for active tuberculosis, and nine (8.8%) were undernourished. The median hemoglobin was 10.1 g/dL (IQR 8.8–11.3 g/dL). Forty-two women (41.2%) reported drinking from 1 to 40 alcoholic beverages per week, thirty-four women (33.3%) used some form of hormonal contraception, and eight women (7.8%) reported smoking from 1 to 10 cigarettes per day.

Overall, these women contributed 94.4 person-years of follow-up after ART initiation, with 95 women (93.1%) remaining at month 6 and 90 (88.2%) at month 12. The median CD4 cell count increased to 236 cells/μL (IQR 159–323 cells/μL) by month 6 and 240 cells/μL (IQR 182–332 cells/μL) by month 12. Plasma HIV-1 RNA was <100 copies/mL for 72.6% of participants at month 6 and for 54.4% at month 12. By a less stringent criterion, plasma HIV-1 RNA was <400 copies/mL for 82.1% of participants at month 6 and for 85.6% at month 12.

### Baseline endothelial activation biomarkers

At baseline, there were significant correlations between several endothelial activation biomarkers and plasma HIV-1 RNA levels: soluble ICAM (ρ = 0.28, p = 0.005), E-selectin (ρ = 0.22, p = 0.02), and VCAM-1 (ρ = 0.32, p = 0.001). The correlation between plasma ANG-2 and plasma HIV-1 RNA was not significant (ρ = 0.19, p = 0.06), but plasma ANG-2 was positively correlated with soluble ICAM (ρ = 0.33, p < 0.001), E-selectin (ρ = 0.32, p < 0.001), and VCAM-1 (ρ = 0.32, p = 0.001). There were no significant correlations between plasma ANG-1 and other endothelial activation biomarkers or plasma HIV-1 RNA. None of the biomarkers tested correlated with the pre-treatment nadir or baseline CD4 count.

### Changes in endothelial activation biomarkers after ART initiation

**Figure 1:**
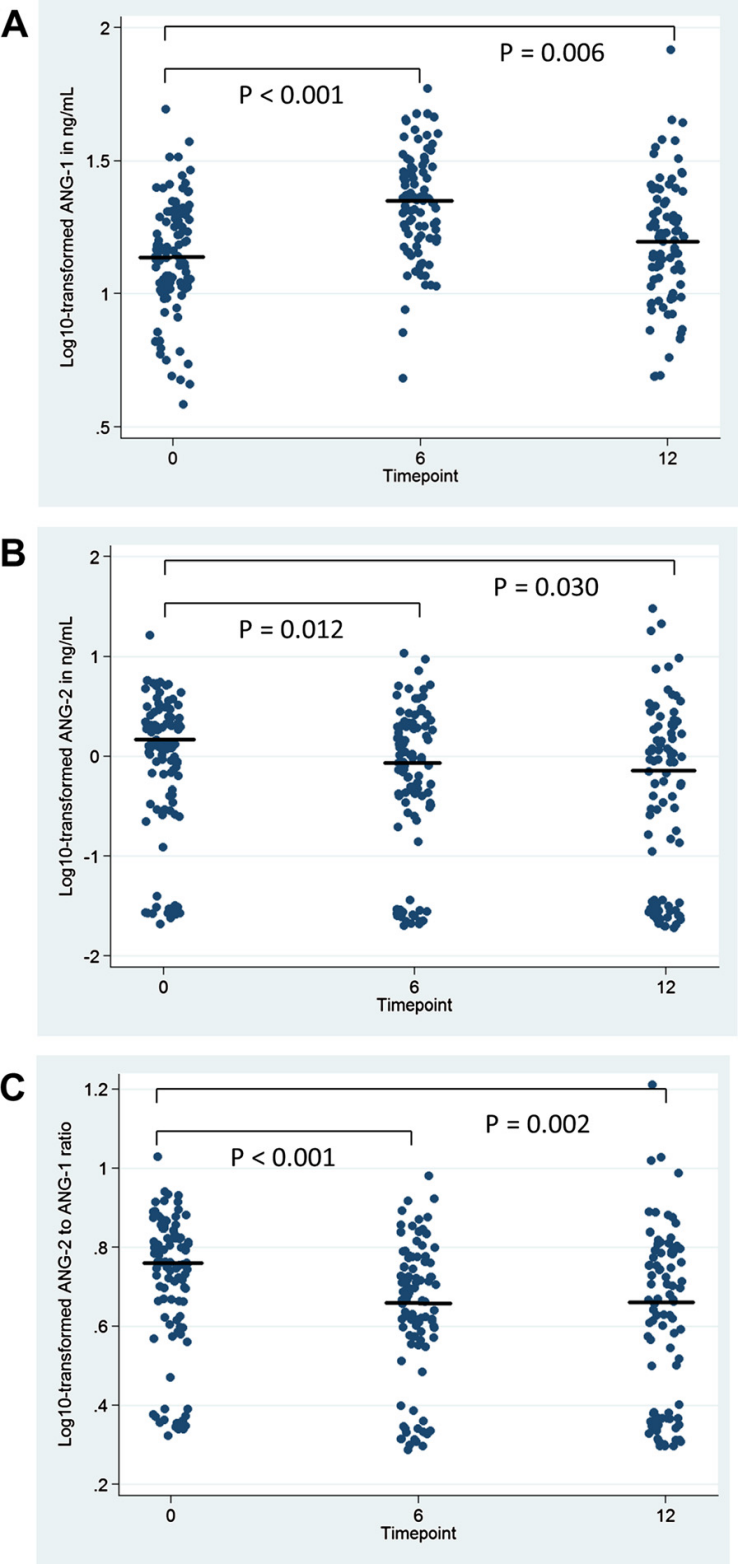
**Log**_**10**_**-transformed Plasma Angiopoietin-1 (A), Angiopoietin-2 (B), and Angiopoietin-2:Angiopoietin-1 Ratio (C) after ART Initiation.** Solid circles represent individual data points. Horizontal lines through the data points represent group median values. P values indicate pairwise comparisons between time-points (baseline, month 6, and month 12 after ART initiation) based on Wilcoxon matched-pairs signed rank tests, as presented in Table 1. There were 95 paired samples for the baseline to month 6 comparison, and 89 paired samples for the baseline to month 12 comparison. ANG-1 = angiopoietin-1, ANG-2 = angiopoietin-2.

Table 1 presents median values for plasma HIV-1 RNA and endothelial activation biomarkers at each time-point, with significant differences indicated. As expected, plasma HIV-1 RNA decreased after ART initiation. Comparing month 6 and month 12 to baseline levels, plasma ANG-1 increased (p < 0.001 for month 6, p = 0.006 for month 12, Figure 1A), while plasma ANG-2 decreased at both time-points (p = 0.01 for month 6, p = 0.03 for month 12, Figure 1B). This resulted in a highly significant decrease of the ANG-2:ANG-1 ratio at both time-points (p < 0.001 for month 6, p = 0.002 for month 12, Figure 1C). In addition, levels of soluble ICAM-1 decreased at both 6 and 12 months after ART initiation (p < 0.001 for both time-points). While soluble E-selectin levels were lower at month 6 than at baseline, they were no different than baseline at month 12. In contrast, soluble VCAM-1 levels were not different at month 6, but decreased at month 12. Plasma ANG-2 levels were detectable in 84.3% of participants at baseline, 81.0% at month 6, and 68.9% at month 12 (p = 0.008 for difference across time-points).

**Table 1:** Comparison between baseline and 6 or 12 months after ART initiation

**Variable**		**Baseline to month 6 comparison **^**a**^		**Baseline to month 12 comparison **^**a,b**^	
	**Baseline median (IQR)**	**Month 6 median (IQR)**	**P value**	**Month 12 median (IQR)**	**P value**
Plasma HIV-1 RNA (copies/mL)	349,066 (149,106–919,173)	<100 (<100–154)	<0.001	<100 (<100–176)	<0.001
CD4 Cell count (cells/μL)	124 (79–164)	236 (159–323)	<0.001	240 (182–332)	<0.001
Angiopoietin-1 (ng/mL)	13.7 (10.2–19.1)	22.5 (16.1–30.2)	<0.001	15.8 (11.6–21.9)	0.006
Angiopoietin-2 (ng/mL)	1.50 (0.37–2.43)	0.86 (0.31–1.92)	0.012	0.70 (<0.03–1.59)	0.030
ANG-2/ANG-1 ratio	0.09 (0.02–0.18)	0.03 (0.01–0.08)	<0.001	0.03 (0.003–0.11)	0.002
Soluble ICAM-1 (ng/mL)	397.9 (338.0–489.7)	220.5 (185.2–292.5)	<0.001	255.2 (200.0–312.5)	<0.001
E-Selectin (ng/mL)	24.2 (16.6–32.3)	13.7 (7.4–20.0)	<0.001	25.2 (18.5–30.4)	0.29
VCAM-1 (ng/mL)	763.8 (524.3–1107.6)	762.5 (537.0–1,013.3)	0.63	497.8 (407.2–628.6)	<0.001

*ART* antiretroviral therapy, *IQR* interquartile range.

^a^ Although 90 women completed the study at month 12, there was insufficient sample from one woman for testing of endothelial markers at this time-point. Therefore, there were 95 paired samples for the baseline to month 6 comparison and 89 paired samples for the baseline to month 12 comparison for all variables presented above except plasma HIV-1 RNA (90 paired samples available).

^b^ Differences between angiopoietin-1, E-selectin, and VCAM-1 levels between month 6 and month 12 were all significant at p < 0.001.

### Predictors of change in log_10_-transformed plasma ANG-1 and ANG-2 after ART initiation

Mean log_10_-transformed plasma ANG-1 was 1.13 (standard deviation [SD] 0.21) at baseline and 1.20 (SD 0.22) at month 12, for an overall mean change of 0.07 (SD 0.25). The geometric mean change in plasma ANG-1 at month 12 was an increase of 1.16 ng/mL (SD 1.79 ng/mL). One participant had undetectable plasma ANG-1 at month 6, a result several standard deviations below the log_10_-transformed mean and all other log_10_-transformed ANG-1 values; data from this visit were excluded from GEE analysis. After adjustment for baseline ANG-1, change in plasma ANG-1 after ART initiation was associated at p < 0.10 with hormonal status, nutritional status, Karnofsky performance category, WHO stage, and CD4 category at baseline, and with hormonal status and nutritional status during follow-up (Table 2). In multivariable modeling, higher plasma ANG-1 levels after ART initiation were associated with baseline Karnofsky score <80 and with HIV-1 RNA suppression and higher plasma ANG-2 levels during follow-up. Plasma ANG-1 levels were lower among women who were severely malnourished at baseline and those who became pregnant during follow-up. There were no associations between change in log_10_-transformed plasma ANG-1 and pre-treatment nadir or baseline CD4 count as a continuous predictor.

**Table 2:** **Correlates of change in log**_**10 **_**angiopoietin-1 level after ART initiation, GEE analysis**

	**Bivariable model **^**a**^		**Multivariable model **^**b**^	
	**Mean change, ( 95% CI)**	**P value**	**Mean change, ( 95% CI)**	**P value**
*Baseline predictor*				
Hormonal status				
No exogenous hormones	Reference		Reference	
Oral contraceptive pills	−0.049 (−0.172 to 0.073)	0.43	−0.154 (−0.330 to 0.022)	0.09
DMPA	0.059 (−0.003 to 0.121)	0.06	0.028 (−0.055 to 0.110)	0.51
Norplant	0.013 (−0.061 to 0.086)	0.74	0.039 (−0.052 to 0.131)	0.40
Nutritional status				
Adequate nutrition	Reference		Reference	
Mild to moderate malnutrition	−0.089 (−0.198 to 0.020)	0.11	−0.083 (−0.192 to 0.026)	0.14
Severe malnutrition	−0.191 (−0.236 to −0.147)	<0.001	−0.212 (−0.286 to −0.137)	<0.001
Karnofsky Performance Score				
Asymptomatic or mild symptoms (90–100)	Reference		Reference	
Moderate symptoms (80)	0.031 (−0.046 to 0.109)	0.43	0.039 (−0.041 to 0.119)	0.34
Unable to work (<80)	0.100 (0.030 to 0.170)	0.005	0.091 (0.015 to 0.166)	0.02
WHO Stage				
Stage I	Reference			
Stage II	−0.016 (−0.103 to 0.071)	0.72		
Stage III	−0.070 (−0.138 to −0.002)	0.04		
Stage IV	−0.092 (−0.194 to 0.009)	0.08		
CD4 Category (cells/μL)				
<200	Reference			
200–350	0 (−0.088 to 0.087)	0.98		
>350	−0.038 (−0.070 to −0.007)	0.02		
*Time-dependent predictor*				
Change in Log_10_ Angiopoietin-2 (ng/mL)	0.037 (−0.016 to 0.091)	0.17	0.053 (0.018 to 0.087)	0.003
Suppressed Plasma HIV-1 RNA	0.042 (−0.015 to 0.098)	0.15	0.055 (0 to 0.109)	0.05
Hormonal status				
No exogenous hormones	Reference		Reference	
Oral contraceptive pills	−0.013 (−0.200 to 0.173)	0.89	0.044 (−0.183 to 0.271)	0.70
DMPA	0.053 (−0.011 to 0.117)	0.10	0.020 (−0.065 to 0.105)	0.65
Norplant	0.024 (−0.068 to 0.115)	0.61	—	
Pregnant	−0.200 (−0.371 to −0.029)	0.02	−0.275 (−0.469 to −0.082)	0.005
Nutritional status				
Adequate nutrition	Reference			
Mild to moderate malnutrition	0.044 (−0.151 to 0.238)	0.63		
Severe malnutrition	−0.093 (−0.124 to −0.062)	<0.001		

*CI* Confidence interval, *GEE* generalized estimating equations.

^a^ Adjusted for baseline log_10_ angiopoietin-1 level (ng/mL).

^b^ Adjusted for baseline log_10_ angiopoietin-1 level (ng/mL) and other covariates presented. Change in ANG-2 and virologic suppression were included a priori in multivariable modeling.

N.B. Variables not significant at p < 0.10 in bivariate analysis are not included in the table, including baseline ANG-2, baseline plasma HIV-1 RNA, baseline age, baseline tuberculosis, tobacco use, alcohol use, time-dependent ANG-2, time-dependent plasma HIV-1 RNA, change in plasma HIV-1 RNA, time-dependent Karnofsky Performance Score, time-dependent tuberculosis, and time-dependent CD4 category. Data from one visit with an outlier value for log_10_ angiopoietin-1 have been excluded, as described in the text.

Mean log_10_-transformed plasma ANG-2 was −0.11 (SD 0.72) at baseline and −0.42 (SD 0.89) at month 12, for a mean change of −0.25 (SD 1.04). The geometric mean change at month 12 was a decrease of 0.56 ng/mL (SD 10.91 ng/mL). Table 3 presents the GEE analysis of factors associated with change in log_10_ plasma ANG-2 after ART initiation. After adjustment for baseline ANG-2, change in plasma ANG-2 was associated at p < 0.10 with hormonal status, nutritional status, WHO stage, and CD4 category at baseline, and with plasma ANG-1 (or change in ANG-l), plasma HIV-1 RNA (or change in RNA), hormonal status, nutritional status, tuberculosis, and CD4 category during follow-up. In multivariable modeling, higher plasma ANG-2 was associated with use of oral contraceptive pills at baseline, and higher plasma ANG-1, pregnancy, severe malnutrition, and tuberculosis during follow-up. Lower plasma ANG-2 levels after ART initiation were associated with Stage IV disease at baseline and mild to moderate malnutrition during follow-up. Although virologic suppression was associated with somewhat lower plasma ANG-2 levels, this finding was not significant (mean difference −0.18 log_10_ with virologic suppression, p = 0.07). If plasma HIV-1 RNA was substituted for virologic suppression in this model, higher plasma HIV-1 RNA was associated with higher plasma ANG-2 levels (mean difference 0.13 log_10_ with each increase in log_10_ plasma HIV-1 RNA, p = 0.01) and other findings were very similar (Multivariable Model 2). There were no associations between change in log_10_-transformed plasma ANG-2 and pre-treatment nadir or baseline CD4 count as a continuous predictor.

**Table 3:** **Correlates of change in Log**_**10**_**-transformed angiopoietin-2 level after ART initiation, GEE analysis**

	**Bivariable model **^**a**^		**Multivariable model 1 **^**b**^		**Multivariable model 2 **^**c**^	
	**Mean change (95% CI)**	**P value**	**Mean change (95% CI)**	**P value**	**Mean change (95%)**	**P value**
*Baseline predictor*						
Hormonal status						
No exogenous hormones	Reference		Reference		Reference	
Oral contraceptive pills	0.474 (0.175 to 0.772)	0.002	0.661 (0.393 to 0.929)	<0.001	0.660 (0.397 to 0.922)	<0.001
DMPA	0.166 (−0.080 to 0.411)	0.19	0.078 (−0.152 to 0.307)	0.51	0.082 (−0.144 to 0.308)	0.48
Norplant	−0.290 (−0.960 to 0.380)	0.40	−0.389 (−1.012 to 0.235)	0.22	−0.396 (−0.997 to 0.205)	0.20
Nutritional status						
Adequate nutrition	Reference					
Mild to moderate malnutrition	−0.278 (−0.670 to 0.113)	0.16				
Severe malnutrition	−0.625 (−0.761 to −0.489)	<0.001				
WHO stage						
Stage I	Reference		Reference		Reference	
Stage II	0.031 (−0.344 to 0.407)	0.87	−0.088 (−0.439 to 0.263)	0.62	−0.061 (−0.413 to 0.291)	0.73
Stage III	−0.151 (−0.466 to 0.163)	0.35	−0.307 (−0.621 to 0.006)	0.06	−0.291 (−0.604 to 0.022)	0.07
Stage IV	−0.546 (−0.998 to −0.093)	.02	−0.492 (−0.946 to −0.039)	.03	−0.444 (−0.892 to 0.004)	0.05
CD4 Category (cells/μL)						
<200	Reference					
200–350	−0.260 (−0.515 to −0.006)	0.04				
>350	0.060 (−0.081 to 0.202)	0.40				
*Time-dependent predictor*						
Log_10_ Angiopoietin-1 (ng/mL)	0.683 (0.144 to 1.222)	0.01				
Change in Log_10_ ANG-1 (ng/mL)	0.709 (0.134 to 1.284)	0.02	0.953 (0.536 to 1.370)	<0.001	0.944 (0.534 to 1.354)	<0.001
Suppressed Plasma HIV-1 RNA	−0.158 (−0.381 to 0.064)	0.16	−0.182 (−0.378 to 0.014)	0.07		
Log_10_ Plasma HIV-1 RNA (copies/mL)	0.142 (0.050 to 0.235)	0.003			0.128 (0.029 to 0.228)	0.012
Change in Log_10_ Plasma HIV-1 RNA (copies/mL)	0.102 (0.022 to 0.182)	0.01				
Hormonal status						
No exogenous hormones	Reference		Reference		Reference	
Oral contraceptive pills	0.367 (0.130 to 0.604)	0.002	0.019 (−0.160 to 0.199)	0.83	0.042 (−0.127 to 0.210)	0.63
DMPA	0.264 (0.021 to 0.506)	0.03	0.098 (−0.130 to 0.326)	0.40	0.123 (−0.108 to 0.353)	0.30
Norplant	−0.353 (−1.090 to 0.383)	0.35	—		—	
Pregnant	1.152 (0.591 to 1.712)	<0.001	1.302 (0.795 to 1.810)	<0.001	1.299 (0.749 to 1.849)	<0.001
Nutritional status						
Adequate nutrition	Reference		Reference		Reference	
Mild to moderate malnutrition	−0.185 (−0.583 to 0.213)	0.36	−0.325 (−0.615 to −0.035)	0.03	−0.353 (−0.664 to −0.042)	0.03
Severe malnutrition	0.627 (0.441 to 0.813)	<0.001	0.688 (0.406 to 0.970)	<0.001	0.729 (0.433 to 1.015)	<0.001
Current tuberculosis	0.635 (0.191 to 1.079)	0.005	0.557 (0.137 to 0.977)	0.009	0.491 (0.053 to 0.929)	0.03
CD4 Category (cells/μL)						
<200	Reference					
200–350	−0.266 (−0.526 to −0.007)	0.04				
>350	−0.145 (−0.435 to 0.145)	0.33				

*CI* Confidence interval, *GEE* generalized estimating equations.

^a^ Adjusted for baseline log_10_ angiopoietin-2 level (ng/mL).

^b^ Adjusted for baseline log_10_ angiopoietin-2 level (ng/mL) and other covariates presented. Change in ANG-1 and virologic suppression were included a priori in multivariable modeling.

^c^ Adjusted for baseline log_10_ angiopoietin-2 level (ng/mL) and other covariates presented. Change in ANG-1 was included a priori and log_10_ plasma HIV-1 RNA was substituted for virologic suppression in multivariable modeling.

N.B. Variables not significant at p < 0.10 in bivariate analysis are not included in the table, including baseline ANG-1, baseline plasma HIV-1 RNA, baseline age, baseline Karnofsky Performance Score, baseline tuberculosis, tobacco use, alcohol use, and time-dependent Karnofsky Performance Score. Data from one visit with an outlier value for log_10_ angiopoietin-1 have been excluded, as described in the text.

### Mortality after ART initiation

Five women died during follow-up, for a mortality rate of 5.3 per 100 (95% confidence interval [CI] 2.2–12.7) person-years of observation. The deaths occurred from 78 to 313 days after ART initiation. All five deaths occurred during hospitalizations for acute illness, but the precise cause of death was unknown and autopsies were not performed. All women who died had adequate nutritional status at baseline by BMI criteria, and WHO stage varied (one stage I, one stage II, two stage III with tuberculosis, and one stage IV with Kaposi’s sarcoma). Four of these women had CD4 counts <100 cells/μL, and four had hemoglobin values <9.0 g/dL; all met at least one of these criteria.

### Predictors of mortality

There were no significant differences in plasma levels of soluble ICAM, VCAM, or E-selectin between women who died and those who did not (p = 0.07, p = 0.27, p = 0.27, respectively). Although baseline plasma ANG-1 levels were not significantly different (median 9.85 ng/mL [IQR 9.14–16.76] versus median 13.80 ng/mL [IQR 10.56–19.12 ng/mL, p = 0.46), baseline plasma ANG-2 levels were significantly higher among women who died after ART initiation than among women who did not (median 2.85 ng/mL [IQR 2.47–5.74 ng/mL] versus median 1.32 ng/mL [IQR 0.35–2.18 ng/mL], p = 0.01). In addition, the ANG-2:ANG-1 ratio was higher among women who died than among women who did not (median 0.27 [IQR 0.27–0.29] versus median 0.08 [IQR 0.02–0.16], p = 0.005). All five women who died were in the top quartile of ANG-2:ANG-1 ratio values.

Table 4 presents the Cox proportional hazards regression for mortality after ART initiation. In univariate analysis, hemoglobin, log_10_ plasma ANG-2, log_10_ ANG-2:ANG-1 ratio, and log_10_ soluble ICAM-1 were associated with mortality at p < 0.10. No predictors remained significant in a model including hemoglobin, log_10_ plasma ANG-2, and log_10_ soluble ICAM-1. Because soluble ICAM-1 was correlated with both hemoglobin (ρ = −0.24, p = 0.01) and plasma ANG-2 levels (ρ = 0.33, p = 0.008) but hemoglobin and plasma ANG-2 were not correlated (ρ = −0.02, p = 0.84), we also evaluated a model including only hemoglobin and log_10_ plasma ANG-2. In this model, mortality risk after ART initiation was related to lower baseline hemoglobin (adjusted relative hazard [aRH] 0.41, 95% CI 0.18–0.92 for each g/dL) and higher baseline plasma ANG-2 (aRH 75.06, 95% CI 1.98–2,846 for each log_10_ ng/mL). In a sensitivity analysis in which women who were lost to follow-up were assumed to have died, log_10_ plasma ANG-2 was still associated with an increased risk of mortality (aHR 8.43, 95% CI 1.42–50.07), but hemoglobin was not (aHR 0.77, 95% CI 0.51–1.16).

**Table 4:** Predictors of mortality following ART initiation

**Predictor at ART initiation**	**HR, 95% CI**	**P value**	**Model 1 adjusted HR, 95% CI**	**P value**	**Model 2 Adjusted HR, 95% CI**	**P value**
Age (years)	1.04 (0.90 − 1.21)	0.57				
BMI (kg/m^2^)	0.94 (0.78–1.14)	0.56				
Karnofsky Performance Scale						
Minor symptoms (90)	Reference					
Moderate symptoms (80)	1.11 (0.11–10.63)	0.93				
Unable to work (<80)	5.34 (0.55–51.38)	0.15				
Current tuberculosis	3.01 (0.50–18.02)	0.23				
WHO Stage						
Stage I	Reference					
Stage II	0.66 (0.04–10.49)	0.77				
Stage III	0.85 (0.08–9.34)	0.89				
Stage IV	1.80 (0.11–28.77)	0.68				
CD4 Cell count (cells/μL)	0.99 (0.97–1.01)	0.22				
Log_10_ Plasma HIV-1 RNA (copies/mL)	3.00 (0.62–14.60)	0.17				
Hemoglobin (g/dL)	0.59 (0.33–1.07)	0.08	0.51 (0.21–1.23)	0.13	0.41 (0.18–0.92)	0.03
Log_10_ Angiopoietin-1 (ng/mL)	0.25 (0.005–13.63)	0.50				
Log_10_ Angiopoietin-2 (ng/mL)	18.85 (1.52–233.7)	0.02	41.31 (0.82–2,086)	0.06	75.06 (1.98–2,846)	0.02
Log_10_ ANG-2/ANG-1 ratio	19.99 (1.66–240.5)	0.02				
Log_10_ s-ICAM (ng/mL)	10,062 (18.29–5,536,091)	0.004	355.8 (0.22–581,538)	0.12		
Log_10_ E-selectin (ng/mL)	11.17 (0.18–696.4)	0.25				
Log_10_ VCAM (ng/mL)	9.33 (0.14–644.0)	0.30				

*ART* antiretroviral therapy, *CI* confidence interval, *HR* hazard ratio.

## Discussion

This study adds to the very limited data on angiopoietins in HIV-1 infection and, to our knowledge, demonstrates their clinical significance in this setting for the first time. The women we studied had advanced HIV disease, with high plasma HIV-1 RNA and low CD4 cell counts at ART initiation. The majority achieved virologic suppression after ART. We found that plasma ANG-1 increased and plasma ANG-2 decreased after ART initiation, with a concomitant decline in the plasma ANG-2:ANG-1 ratio. These changes were accompanied by consistent decreases in soluble ICAM-1, and, less consistently, reductions in plasma levels of soluble VCAM-1 and E-selectin. Such decreases in plasma levels of soluble adhesion molecules are in accordance with the published literature [10,11].

Plasma ANG-1 levels were lower in women with severe malnutrition at baseline and pregnancy during follow-up. Higher plasma ANG-2 levels were associated with oral contraceptive pill use at baseline and with pregnancy, severe malnutrition, and tuberculosis during follow-up. In addition, higher plasma ANG-2 levels after ART initiation were associated with higher plasma HIV-1 RNA. Baseline ANG-2 levels and ANG-2:ANG-1 ratio values were higher in women who died after ART initiation. As previously demonstrated in another cohort [12], baseline soluble ICAM-1 levels were associated with an increased risk of mortality over follow-up. We found that soluble ICAM-1 levels correlated significantly with both plasma ANG-2 and hemoglobin, and that a model including only plasma ANG-2 and hemoglobin fit our data better than one including all three predictors.

Peripheral blood (plasma and serum) ANG-2 levels, which are indicative of an activated endothelium, are often below the limit of assay detection in healthy persons. In contrast, 84% of participants in this study had detectable plasma ANG-2 levels at baseline. While plasma ANG-2 levels were linked to opportunistic illness (i.e., tuberculosis and very low BMI) in this population, the decrease in the plasma ANG-2:ANG-1 ratio after ART initiation and the association between plasma ANG-2 levels and HIV-1 RNA levels after ART suggest that HIV-1 itself can drive endothelial activation. In support of our hypothesis that ART would not completely normalize endothelial activation, plasma ANG-2 was detectable in 69% of participants at month 12. These findings, as well as the association of higher plasma ANG-2 levels with increased mortality, require confirmation in other study populations. In addition, because our study was not aimed at elucidating how angiopoietin levels are regulated in HIV-1 infection, further work is needed to demonstrate whether plasma ANG-2 is increased primarily by locally induced cytokines, by a direct effect of HIV-1 on endothelial cells [25], or by other factors. Of note, circulating monocytes are a site of ANG-2 production [26] and are activated in HIV-1 infection. Increased levels of soluble CD14, which is expressed mainly by macrophages and may be a consequence of microbial translocation [27], have also been related to mortality in HIV-1-infected patients [28]. Because inflammation from opportunistic infections may also increase plasma HIV-1 RNA levels, it is possible that the association we detected between HIV-1 RNA and endothelial activation was due primarily to immune reconstitution and undiagnosed opportunistic infections in this population.

We detected significant associations between angiopoietin levels and hormonal status, with higher plasma ANG-2 levels in pregnancy and with the use of oral contraceptive pills. ANG-2 is known to play an essential role in embryonic angiogenesis and placental development [29], and therefore its association with pregnancy is not surprising. We could find no previous report of an association between angiopoietin levels and hormonal contraception. However, estrogen stimulates ANG-2 mRNA expression and inhibits ANG-1 expression in non-reproductive organs [30], and plasma ANG-2 levels are higher in non-pregnant women than in men [31]. We also found a relationship between angiopoietin levels and severe malnutrition as defined by BMI < 16. Although there is evidence that poor dietary choline intake increases ANG-2 in an animal model [32], we could identify no studies investigating the effect of nutritional status on plasma angiopoietin levels in adults. Future studies of angiopoietin biology in female participants and in populations in which malnutrition is prevalent should consider adjustments for these factors.

Because endothelial cells are involved in many aspects of vascular biology, including barrier function, immune surveillance, blood clotting, and atherosclerosis, ongoing endothelial activation as manifested by persistent elevations in plasma ANG-2 may predispose to a wide array of adverse outcomes. Since the SMART (Strategies for Management of Antiretroviral Therapy) trial demonstrated a high rate of mortality in untreated HIV-1 infection due to non-AIDS events, numerous biomarkers of adverse outcomes have been identified [27]. In addition, the question of whether adjunct treatments used in combination with ART can reduce HIV-1-related inflammation has been posed [3,33]. Although anti-inflammatory drugs have some effect on inflammatory biomarkers [34], interventions that target inflammatory biomarkers in HIV-1 infection have largely been unsuccessful to date [35-37]. ANG-2 is an important mediator of endothelial activation that has been linked to reduced endothelial nitric oxide availability and vascular dysfunction [38,39], and may be a point of intervention to modulate host response. If further research confirms our findings in this study, investigation of whether HIV-1-related endothelial activation predicts vascular dysfunction and end-organ disease may lead to the development of new intervention strategies. Therapeutic agents targeting the angiopoietin/Tie-2 system, ANG-2 antagonists, and agents that increase endothelial nitric oxide are all in development [13,40], and may have potential applications in HIV-1-infected persons with elevated plasma ANG-2 levels.

An important strength of this study is its prospective design, in which participants were used as their own controls. However, this study had several limitations. First, a limited quantity of stored heparinized plasma was available for this study, and all potential biomarkers of interest could not be tested. Second, our limited sample size and follow-up reduced power and precision for our survival analysis. Nevertheless, we detected a significant association between plasma ANG-2 levels and mortality. Third, we could not establish causes of death and were unable to control for specific opportunistic infections other than tuberculosis, due to limited diagnostic capability at the field site. Fourth, outcomes for the women who were lost to follow-up are unknown. Because loss to follow-up may have been associated with mortality, we conducted a sensitivity analysis and found that plasma ANG-2 levels remained associated with mortality when these women were assumed to have died. Fifth, no clinically relevant cut-points have been established for endothelial activation biomarkers, and assays are not standardized between laboratories, making direct comparisons between studies difficult to interpret. Finally, our analysis was restricted to a cohort of Kenyan women with advanced HIV-1 disease and opportunistic infections. Although many adult patients in Africa present with a similar degree of immunosuppression, our findings cannot be generalized to men, to adults in other settings, or to adults with less advanced HIV-1 disease. Additional research will be needed to determine if angiopoietin levels are influenced by HIV-1 acquisition and if biomarkers of endothelial activation remain associated with adverse outcomes among HIV-1-infected patients taking long-term suppressive ART.

## Conclusions

In conclusion, endothelial activation was present in women with advanced HIV infection, and improved after ART initiation, with an increase in plasma ANG-1 levels and a decrease in plasma ANG-2 levels. Higher plasma ANG-2 levels were associated with higher plasma HIV-1 RNA after treatment initiation. Angiopoietin dysregulation was associated with pregnancy, malnutrition, and tuberculosis, and may also have been related to undiagnosed immune reconstitution inflammatory syndrome or opportunistic infections. Baseline plasma ANG-2 levels were higher among women who died after ART initiation. Our results add significantly to the limited data on angiopoietin levels in HIV-1-infected patients, and suggest that further investigation into HIV-1-related endothelial activation is warranted, including research on whether plasma ANG-1 and ANG-2 may be useful biomarkers for HIV-1-related morbidity and mortality.

## Competing interests

WCL is listed as a co-inventor on a patent applied for by the University Health Network (Toronto, ON, Canada) to develop point-of-care tests for endothelial activation biomarkers in infectious diseases. All other authors report no conflict of interest.

## Authors’ contributions

Conceived and designed the experiments: SMG RSM JO WCL. Performed or supervised field work: SMG WJ BBAE RSM. Performed or supervised laboratory testing: NR WCL JO. Analyzed the data: SMG KAT. Wrote the paper: SMG. All authors read and approved the final manuscript: SMG NR KAT WJ BBAE RSM JO WCL.

## Pre-publication history

The pre-publication history for this paper can be accessed here:

http://www.biomedcentral.com/1471-2334/13/263/prepub
